# Monoamine Oxidase Inhibition by Major Tanshinones from *Salvia miltiorrhiza* and Selective Muscarinic Acetylcholine M_4_ Receptor Antagonism by Tanshinone I

**DOI:** 10.3390/biom11071001

**Published:** 2021-07-08

**Authors:** Ritu Prajapati, Se Eun Park, Su Hui Seong, Pradeep Paudel, Fazlin Mohd Fauzi, Hyun Ah Jung, Jae Sue Choi

**Affiliations:** 1 Department of Food and Life Science, Pukyong National University, Busan 48513, Korea; ritpraz@gmail.com (R.P.); gogo1685@mail.ulsan.ac.kr (S.E.P.); shseong@hnibr.re.kr (S.H.S.); ppradeep@olemiss.edu (P.P.); 2 Department of Biomedical Science, Asan Medical Institute of Convergence Science and Technology, University of Ulsan, Seoul 05505, Korea; 3 Natural Product Research Division, Honam National Institute of Biological Resource, Mokpo 58762, Korea; 4 National Center for Natural Products Research, Research Institute of Pharmaceutical Science, The University of Mississippi, Oxford, MS 38677, USA; 5 Department of Pharmacology and Chemistry, Faculty of Pharmacy, Universiti Teknologi MARA, Puncak Alam 42300, Malaysia; fazlin5465@uitm.edu.my; 6 Department of Food Science and Human Nutrition, Jeonbok National University, Jeonju 54896, Korea

**Keywords:** tanshinone I, Parkinson’s disease, monoamine oxidase inhibition, molecular docking, M_4_ receptor antagonist

## Abstract

Monoamine oxidases (MAOs) and muscarinic acetylcholine receptors (mAChRs) are considered important therapeutic targets for Parkinson’s disease (PD). Lipophilic tanshinones are major phytoconstituents in the dried roots of *Salvia miltiorrhiza* that have demonstrated neuroprotective effects against dopaminergic neurotoxins and the inhibition of MAO-A. Since MAO-B inhibition is considered an effective therapeutic strategy for PD, we tested the inhibitory activities of three abundant tanshinone congeners against recombinant human MAO (hMAO) isoenzymes through in vitro experiments. In our study, tanshinone I (**1**) exhibited the highest potency against hMAO-A, followed by tanshinone IIA and cryptotanshinone, with an IC_50_ less than 10 µM. They also suppressed hMAO-B activity, with an IC_50_ below 25 µM. Although tanshinones are known to inhibit hMAO-A, their enzyme inhibition mechanism and binding sites have yet to be investigated. Enzyme kinetics and molecular docking studies have revealed the mode of inhibition and interactions of tanshinones during enzyme inhibition. Proteochemometric modeling predicted mAChRs as possible pharmacological targets of **1**, and in vitro functional assays confirmed the selective M_4_ antagonist nature of **1** (56.1% ± 2.40% inhibition of control agonist response at 100 µM). These findings indicate that **1** is a potential therapeutic molecule for managing the motor dysfunction and depression associated with PD.

## 1. Introduction

Parkinson’s disease (PD) is the second-most prevalent age-dependent neurodegenerative disease (NDD) and is characterized by the progressive degeneration of dopaminergic and nondopaminergic systems in the substantia nigra, the striatal deficiency of dopamine (DA), and the intracellular aggregation of α-synuclein. It affects nearly 2% to 3% of the elderly population aged over 65 years and presents as motor dysfunctions such as tremor, rigidity, bradykinesia, and postural instability. Besides motor symptoms, nonmotor features such as cognitive impairment, depression, dysautonomia, and other social and behavioral abnormalities exacerbate the disease condition [1,2,3]. Current PD treatment is symptomatic and focused on targeting the dopaminergic system. Levodopa (l-dopa) remains the principal drug used for replenishing reduced DA levels in PD, while adjunct therapeutics include catechol-O-methyl transferase (COMT) inhibitors such as carbidopa, monoamine oxidase B (MAO-B) inhibitors such as selegiline and safinamide, and DA receptor agonists such as pramipexole and apomorphine. Although dopaminergic therapies offer considerable benefits, l-dopa-induced dyskinesia (LID) and motor fluctuation and l-dopa-resistant or nondopaminergic motor features, as well as nonmotor complaints, demand the use of nondopaminergic treatment options. For example, the *N*-methyl-d-aspartate (NMDA) receptor antagonist amantadine is used clinically for LID during PD treatment. Other nondopaminergic neurotransmitters and neuromodulatory systems, such as glutamatergic, serotonergic, cholinergic, histaminergic, cannabinoid, and non-adrenergic, influence the basal ganglia circuit and are also the targets of ongoing studies to enhance the treatment of PD and alleviate the side effects of DA replacement therapy [1,4].

Monoamine oxidases (MAOs, EC 1.4.3.4) are the mitochondria-bound flavoenzymes responsible for the catalytic degradation of biogenic amines, including monoaminergic neurotransmitters, dietary amines, and xenobiotics in the brain and peripheral tissues. In the human brain, MAOs occur in two isoenzyme forms, MAO-A and MAO-B, which are approximately 72% identical in amino acid sequence makeup but vary in substrate preferences, inhibitor specificities, cell and tissue distribution, and immunological properties. MAO-A selectively deaminates serotonin (5-HT), whereas MAO-B preferentially degrades benzylamine and 2-phenylethylamine. DA, noradrenaline, and adrenaline are the common substrates for both isoforms of MAOs. Thus, the inhibitors of MAOs are considered as prophylactic and therapeutic agents for NDDs such as PD, Alzheimer’s disease (AD), schizophrenia, anxiety, and depression. These conditions are characterized by elevated levels and activities of MAOs, which lead to decreased levels of neurotransmitters, increased oxidative stress resulting from the oxidoreductase activity of the enzymes, and the subsequent degeneration of neurons [3,5,6,7].

Acetylcholine (ACh) is a prime neurotransmitter in the striatum released by innervated cholinergic interneurons. Cholinergic transmission is proposed to play a critical role in regulating the local circuits of the striatal complex and the modulation of presynaptic DA release [8,9]. ACh acts through two types of receptors—nicotinic receptors (nAChRs), which are ligand-gated ion channels, and muscarinic receptors (mAChRs), which are G-protein-coupled receptors (GPCRs). Even though both of these receptors have significant roles in the peripheral nervous system and central nervous system (CNS), mAChRs outnumber nAChRs in the CNS and are involved in regulating the neuronal activity and neurotransmitter releases in different areas of the brain to maintain neuronal plasticity and regulate multiple motor and cognitive functions. There are five subtypes of mAChRs: Gq-coupled M_1_, M_3_, and M_5_, which activate phospholipase C and regulate intracellular calcium mobilization, and Gi/o-coupled M_2_ and M_4_, which inhibit adenylyl cyclase, causing a reduction in the cAMP levels [10,11]. M_1_, M_2_, and M_4_ are the predominant mAChRs in the human brain. Quantitative analyses such as immunohistochemical and radioligand-binding studies, which were performed to determine the mAChR distribution, showed that the M_1_, M_2_, and M_4_ receptors are the highly expressed muscarinic receptor subtypes in the frontal, temporal, parietal, and occipital cortices. The M_1_ and M_4_ receptors are highly localized in the hippocampus and the basal ganglia, while the M_2_, M_3_, and M_5_ receptors predominate in the thalamus, peripheral vasculature, and cerebrovasculature, respectively [12,13]. Concerning their location, relative abundance, and neuronal function, mAChRs have been implicated in different psychiatric and neurological disorders, mainly AD, PD, and schizophrenia [11,14].

The dried roots of *Salvia miltiorrhiza*, known as Dansen in traditional Chinese medicine (TCM), have been extensively used as a traditional medicine for various diseases, such as coronary heart diseases, cerebrovascular diseases, AD, PD, renal deficiency, cancer, hepatocirrhosis, and bone loss, either as a single herb or in combination with other herbal medicines. *Salvia miltiorrhiza* is a perennial plant widely distributed in China, Japan, and Korea [15,16]. Compared to the other parts of the plants, the roots of *Salvia* constitute a high amount of phenolic acids, flavonoids, terpenes, and tanshinones [17]. The major bioactive constituents of Dansen include hydrophilic phenolic acids such as salvianolic acids and hydrophobic diterpenoid quinones such as tanshinones [16]. Salvianolic acids A and B have been reported to have protective effects against liver, pulmonary, and renal fibrosis and have an antiproliferative effect on breast cancer cells, head and neck squamous carcinoma cells, and pulmonary and hepatic carcinoma cells [18]. Likewise, lipophilic tanshinones were found to possess a cytotoxic effect on tumor cell lines and anti-inflammatory, antioxidant, angiogenic, and neuroprotective effects [19].

Recent pharmacological studies focusing on the neuroprotective roles of *S*. *miltiorrhiza* have demonstrated the potential activities of its constituents in NDDs, including AD and PD. Phytoconstituents from this species exhibit the inhibition of acetylcholinesterase (AChE), butyrylcholinesterase (BChE), and β-secretase (BACE1), which are implicated in the pathogenesis of AD [16,20,21]. Tanshinone I (**1**) showed a remarkable suppression of the proinflammatory M1 factors—namely, nitric oxide (NO), tumor necrosis factor (TNF)-α, interleukin (IL)-1β, and IL-6 expressed in activated microglia. Further, it enhanced the motor function in vivo and protected against MPTP-induced neurodegeneration [22]. Tanshinone IIA (**2**), in multiple experiments, prevented the loss of dopaminergic neurons via different molecular mechanisms [23,24,25]. Treatments with Danshensu or salvianic acid A displayed enhancement of the motor activity and neuroprotection against rotenone-induced Parkinsonism [26]. Overall, these results insinuate that *Salvia* phytochemicals can act as anti-Parkinson agents. Selegiline and rasagiline are the two most well-known MAO-B inhibitors that display the protection of dopaminergic neurons from cell death induced by dopaminergic neurotoxins (MPTP, MPP+, and 6-OHDA); ischemia; excitotoxins; and other insults [3]. Previously, Dittman et al. (2004) evaluated four compounds from *S*. *miltiorrhiza*: **1**, **2**, cryptotanshinone (**3**), and dihydrotanshinone I (Dtan I) for recombinant human MAO-A (hMAO-A) inhibition and found Dtan I as the most active compound, with an IC_50_ value of 23 µM, followed by **1**, **3**, and **2**, with IC_50_ values of 80, 84, and >400 µM, respectively [27]. However, the inhibitory potential against recombinant human MAO-B (hMAO-B) by tanshinones from Danshen has not been evaluated. Moreover, the mechanisms of hMAO-A and hMAO-B inhibition for these compounds are unknown. Thus, in this study, we investigated the recombinant hMAO-A and hMAO-B inhibition potentials of three major tanshinones: **1**, **2**, and **3** isolated from the roots of *S*. *miltiorrhiza*. We examined the enzyme inhibition mode and intermolecular interactions engaged in enzyme inhibition through kinetic experiments and computational docking to elucidate the mechanism of enzyme inhibition. Many natural and synthetic compounds can target more than one locus of insult during pathogenesis, and such multifunctional agents have emerged as master keys or magic bullets in the treatment of multifactorial diseases [28]. Since in silico target prediction aids in determining the polypharmacology of a compound, we used computational proteochemometric modeling (PCM) to identify the most probable protein targets of potent MAO inhibitors. After selecting the most feasible drug target for therapeutic usefulness in PD among the top ten targets predicted for the test compounds, we performed cell and nuclear receptor-based functional GPCR assays and molecular docking to determine the modulatory action on targeted GPCRs and established their pharmacological role in the management of PD.

## 2. Materials and Methods

### 2.1. Chemicals and Reagents

**Figure 1:**
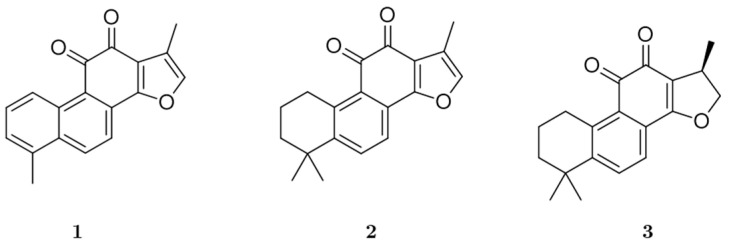
Chemical structures of tanshinone I (**1**), tanshinone IIA (**2**), and cryptotanshinone (**3**) isolated from *Salvia miltiorrhiza*.

Tanshinone I (**1**), tanshinone IIA (**2**), and cryptotanshinone (**3**) of >98% purity were obtained from the dried roots of *Salvia miltiorrhiza*, as described in our published report [29]. The chemical structures of **1**, **2**, and **3** are shown in Figure 1. Recombinant hMAO-A and hMAO-B, l-deprenyl·HCl, clorgyline·HCl, 3-isobutyl-1-methylxanthine (IBMX), and acetylcholine chloride (ACh) were purchased from Sigma Aldrich (Darmstadt, Germany). Dulbecco’s modified Eagle’s medium (DMEM), Hank’s balanced salt solution (HBSS), and 4-(2-hydroxyethyl)-1-piperazineethanesulfonic acid (HEPES) were bought from Invitrogen (Waltham, MA, USA). The remaining reagents and chemicals were purchased from commercial suppliers.

### 2.2. Human Monoamine Oxidase Inhibition Assay

The inhibitory activities on hMAO-A and hMAO-B by three tanshinones: **1**, **2**, and **3** were evaluated by using a MAO-Glo^TM^ chemiluminescent assay kit (Promega, Madison, WI, USA). The experimental procedures for this experiment were as described earlier [30,31]. Briefly, 12.5 µL of the test compound or l-deprenyl/clorgyline.HCl was added to a 12.5-µL aliquot of beetle luciferin derivative substrate (the initial concentrations of hMAO-A and hMAO-B were 160 µM and 16 µM, respectively) in each well of a 96-well plate. An enzyme solution (25 µL) was then added to the test samples to initiate the reaction. After an hour of incubation at 25 °C, a reconstituted luciferin detection reagent (50 µL) was added to each well to stop the reaction and produce a luminescent signal. The final mixture was incubated for an additional 20 min at 25 °C. Then, the luminescence was recorded on a FilterMax F5 Multi-Mode microplate reader (Molecular Devices LLC, San Jose, CA, USA). The amount of luminescence was directly proportional to the residual activity of the hMAO isoenzymes.

### 2.3. hMAO Enzyme Kinetics Experiment

**Figure 2:**
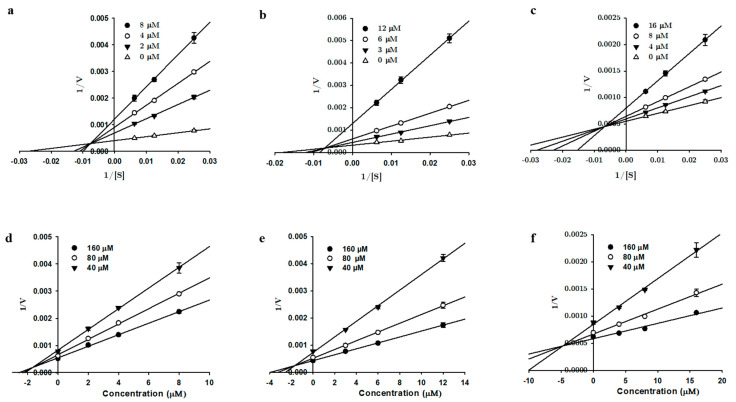
Lineweaver-Burk (**a**–**c**) and Dixon (**d**–**f**) plots of tanshinone I, tanshinone IIA, and cryptotanshinone for hMAO-A inhibition, respectively.

**Figure 3:**
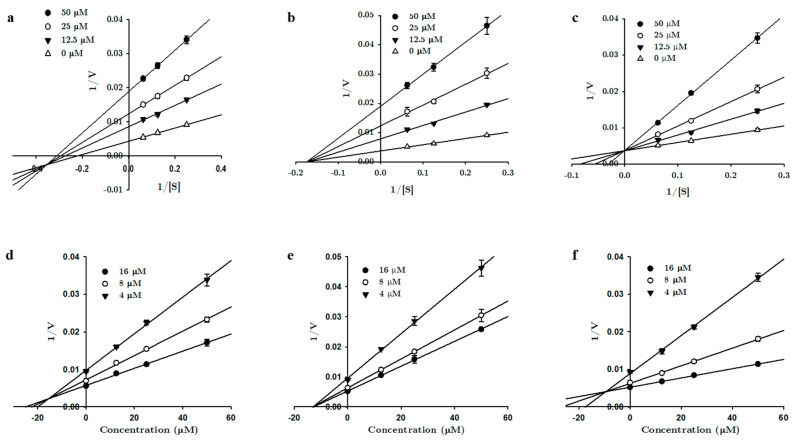
Lineweaver-Burk (**a**–**c**) and Dixon (**d**–**f**) plots of tanshinone I, tanshinone IIA, and cryptotanshinone for hMAO-B inhibition, respectively.

Enzyme inhibition kinetics was performed with tanshinones **1**, **2**, and **3** using varying concentrations of the hMAO substrate (40–160 µM for the hMAO-A kinetics and 4–16 µM for the hMAO-B kinetics). Kinetic experiments were carried out as described by Seong et al. [30] and Paudel et al. [31]. The different concentrations of tanshinones used for the kinetic analyses are presented in Figure 2 and Figure 3. The mode of MAO inhibition was determined from Lineweaver-Burk and Dixon plots, whereas the inhibition constants (*K*_i_) were obtained from secondary plots analyzed using SigmaPlot 12.0 software (SPCC Inc, Chicago, IL, USA).

### 2.4. Prediction of Protein Targets

PCM was used to predict the potential protein targets of **1**, **2**, and **3**. The experimental model for determining the targets was based on a machine learning prototype with a Parzen Rosenblatt window covering 55,079 compounds against 99 human proteins. Representations of the compound molecular characterizations were obtained by extended connectivity fingerprints (ECFP_4) generated using jCompoundMapper. The chemical similarity was calculated using the Aitchison and Aitken kernel function. Protein sequences were aligned using the MUSCLE algorithm created by the bui3d package; comparisons between two protein sequences were made, and the similarities were calculated. Internal and external validation were done for the prediction model, evaluating the sensitivity, specificity, Matthews correlation coefficient, and area under the curve. The detailed PCM was described in an earlier report [32].

### 2.5. In Vitro Functional GPCR Assay

The cellular and nuclear receptor functional assays were carried out at Eurofins Cerep (Le Bois I’ Eveque, France) following the in-house protocols (catalog items G029-1262, G029-1474, G030-1658, G030-1659, G031-1243, G031-1244, G032-1670, G032-1671, G033-4217, and G033-4212). The experimental methods for the assays were as described in the previous reports [33,34,35,36] and the general methodology is summarized in the Supplementary Information Table S1. Recombinant human Chinese hamster ovary (CHO) cells and rat basophilic leukemic (RBL) cells were transfected with the GPCR genes of interest (hM_1_R, hM_2_R, hM_3_R, hM_4_R, and hM_5_R). The functional assay provided the readouts based on the measurements of calcium ion mobilization (for the G_αq/11_-coupled receptors) and cAMP level (for the G_αi/o_-coupled receptors).

#### 2.5.1. Measurement of the cAMP Level

Stable CHO cells expressing the transfected cDNA of human M_2_ receptors were suspended in a medium containing HBSS buffer complemented with 20-mM HEPES buffer (pH 7.4) and 500-µM IBMX, whereas the stable human M_4_ receptor cloned CHO cells were distributed in HBSS buffer with 20-mM HEPES/NaOH (pH 7.4), 70-mM NaCl, 5.33-mM KCl, 1.25-mM CaCl_2_, 0.5-mM MgCl_2_, 0.41-mM MgSO_4_, 0.441-mM KH_2_PO_4_, 0.3-mM Na_2_HPO_4_, 0.1% glucose, and 500-µM IBMX. These cell suspensions were distributed into their respective assay plates at a density of 10^4^ cells/well and incubated for 5 min at 25°C with/without the test compound or standard. After that, NKH 477 was added to make the final concentrations of either 5 µM (for M_2_R) or 1 µM (for M_4_R) and incubated for 10 min at 37 °C. The cells were then lysed, and the fluorescence acceptor (D2-labeled CAMP) and fluorescence donor (anti-CAMP antibody labeled with europium cryptate) were dispensed into the cell plate. The resulting mixture was incubated for the next 1 h at RT. The HTRF reading was subsequently taken using a PerkinElmer Envision microplate reader (Waltham, MA, USA) at an excitation intensity of 337 nm and emission intensities of 620 and 665 nm. The cAMP level was calculated as the ratio of the signal measured at 665 nm to that measured at 620 nm. The agonist and antagonist activities were illustrated as a percentage (%) stimulation of the control agonist response and as a % inhibition of the control agonist response. The agonist effect was determined as a % of the control response to 3-µM and 1-µM acetylcholine for M_2_R and M_4_R, respectively. Likewise, the antagonist behavior was represented as a % of the inhibition of the control response to 0.3-µM and 100-nM acetylcholine for M_2_R and M_4_R, respectively. The reference agonist for the assay was acetylcholine chloride, and the reference antagonists were methoctramine (for M_2_R) and PD 102807 (for M_4_R).

#### 2.5.2. Measurement of the Intracellular Calcium Levels

The calcium ion influx was evaluated fluorometrically to determine the functional activity of **1** on M_1_R, M_3_R, and M_5_R. Stable human M_1_R and M_3_R cloned CHO cells were suspended in DMEM buffer complemented with and without 0.1% delipidated fetal calf serum and dispensed in a microplate at a density of 3 × 10^4^ cells/well and 2.5 × 10^4^ cells/well, respectively. A fluorescent probe (Fluo4 Direct, Invitrogen, Waltham, MA, USA) blended with probenecid and 20-M HEPES (pH 7.4) was applied to each well of cells and equilibrated at 37 °C for 60 min, followed by an additional 15 min at 22 °C. The assay plate was placed in a microplate reader (CellLux, PerkinElmer, Waltham, MA, USA), and the test solution, reference agonist, or HBSS buffer (control) were added to the plate, and ultimately, the fluorescence was measured.

Likewise, hM_5_R-transfected RBL cells were distributed in HBSS buffer containing 20-mM HEPES (pH 7.4) and dispensed into the assay plate at a concentration of 2.0 × 10^4^ cells/well. The fluorescent probe (Fluo8, AAT Bioquest, Sunnyvale, CA, USA) was blended with probenecid in the buffer and added to each well and left to equilibrate for 60 min at 30 °C. The assay plate was placed in a microplate reader (FlipR Tetra, Molecular Device, San Jose, CA, USA), and the test solution, reference agonist, or HBSS buffer (control) were added. The fluorescence was then measured.

The agonist activity was determined as the % of the control response to 100-nM, 1-µM, and 624-nM acetylcholine for M_1_R, M_3_R, and M_5_R, respectively. Likewise, the antagonist behavior was measured as a % inhibition of the control response to 3-nM, 100-nM, and 10-nM acetylcholine for M_1_R, M_3_R, and M_5_R, respectively. The reference antagonist drugs for M_1_R, M_3_R, and M_5_R were pirenzepine, 4-DAMP, and atropine sulfate, respectively.

### 2.6. Molecular Docking Simulation

Autodock4.2 software was used for the molecular docking of compounds **1**, **2**, and **3** to the X-ray crystallographic structures of hMAO-A and hMAO-B acquired from the RCSB Protein Data Bank (PBD) with IDs 2BXR and 2BYB, respectively [37]. The crystal structure of the M_4_ receptor bound to tiotropium with a RCSB PDB ID 5DSG was used for studying the interactions between **1** and the M_4_ receptor. The source of the 3D chemical structures of **1**, **2**, and **3** was the PubChem Compound database (NCBI) with CIDs 114917, 164676, and 160254. Similarly, the crystal structures of harmine, deprenyl, acetylcholine, and tiotropium were also derived from NCBI under CIDs 5280953, 5195, 187, and 5487427, respectively. For docking hMAOs, the water and ligand molecules were eliminated using Discovery Studio (v17.2, Accelrys, San Diego, CA, USA), but the cofactor FAD was retained. AutoDockTool (ADT) was used for adding Kollman charges and polar hydrogens to the cleaned proteins. The 3D structures of the compounds were generated using MarvinSketch (v17.1.30, ChemAxon, Budapest, Hungary). The Gasteiger charges and number of rotatable bonds were computed automatically for the ligands using ADT. The coordinates of the active sites of the proteins were generated using AutoGrid. The number of generic algorithm runs was set to ten, and the other docking parameters were set as the default using AutoDock. The ligand–protein complexes with the least binding energies from their respective populated clusters were selected for the docking analysis. The interactions in the complexes were visualized using Discovery Studio.

### 2.7. Drug-Likeness and ADMET Prediction of Compound ***1***

The pharmacokinetic behaviors, such as human intestinal absorption (HIA), Caco-2 permeability, the blood–brain barrier (BBB) and CNS permeability, and the toxicity profile, were predicted using a web-based pkCSM application [38], whereas the solubility, lipophilicity (Log Po/w*)*, drug-likeness, and lead-likeness of **1** were predicted by SwissADME [39].

## 3. Results

### 3.1. In Vitro Recombinant Human Monoamine Oxidase Inhibition by ***1***, ***2***, and ***3***

The recombinant human monoamine oxidase (hMAO-A and hMAO-B) inhibition potential of the tanshinones **1**, **2**, and **3**, obtained from the roots of *S*. *miltiorrhiza,* were investigated through a chemiluminescent in vitro assay using a MAO-Glo kit. All three tanshinone congeners **1**, **2**, and **3** showed considerable activity against both hMAO-A and hMAO-B (Table 1). Compounds **1**, **2**, and **3** inhibited hMAO-A, with corresponding IC_50_ values of 2.62 ± 0.52, 6.08 ± 0.06, and 8.70 ± 0.06 µM, and also displayed significant hMAO-B inhibition, with the corresponding IC_50_ values of 24.9 ± 3.82, 17.5 ± 0.89, and 23.1 ± 2.10 μM. Bioactive tanshinones were selective toward hMAO-A, **1** being the most potent and selective hMAO-A inhibitor (selectivity index 0.11).

Since compounds **1**, **2**, and **3** are potent MAO inhibitors, we performed an enzyme kinetics study to identify the enzyme inhibition modes and *K*_i_ values. Lineweaver-Burk plots (1/V vs. 1/S) and secondary plots (*K*_mapp_/*V*_maxapp_ and 1/*V*_maxap_*_p_* vs. the inhibitor concentration) were used to determine the kinetic parameters (Figure 2 and Figure 3). The overall results obtained from the kinetics study are tabulated in Table 1. For hMAO-A inhibition, all three compounds exhibited mixed inhibition with the *K*_ic_ < *K*_iu_, suggesting a higher affinity for free enzymes than substrate-bound enzymes. The *K*_ic_ values for **1**, **2**, and **3** were 1.69 ± 0.19, 0.72 ± 0.13, and 4.99 ± 0.34 µM, respectively. With hMAO-B, **1** showed a mixed mode of inhibition, with a *K*_ic_ value of 25.6 ± 1.10 µM and *K*_iu_ value of 17.4 ± 0.78 µM. Compound **2** showed noncompetitive inhibition, with an inhibition constant of 13.7 ± 0.58 µM, and **3** showed competitive enzyme inhibition, with a *K*_i_ value of 9.33 ± 0.10 µM.

### 3.2. Computational Investigation into the Binding Characteristics of Tanshinones to hMAOs

**Figure 4:**
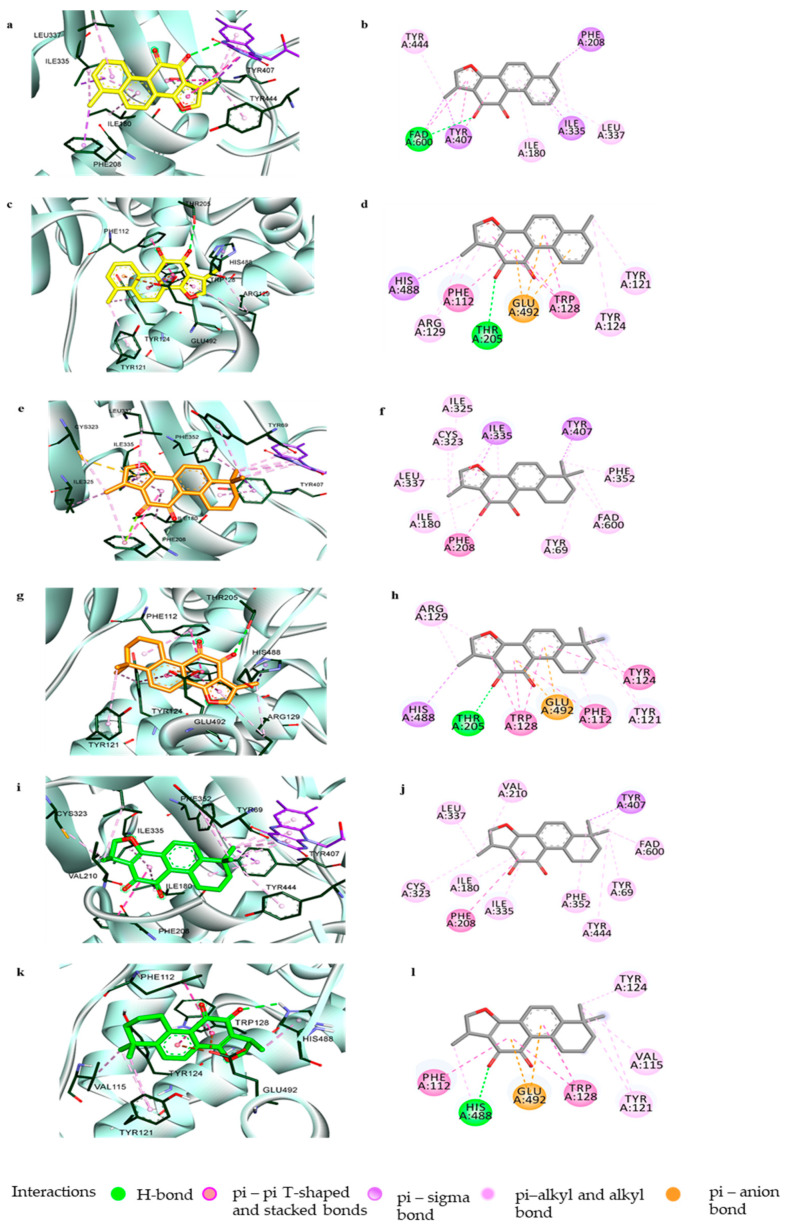
Prediction of the binding modes of the tanshinones with hMAO-A by molecular docking. Close-up view of tanshinone I (yellow), tanshinone IIA (brown), and cryptotanshinone (green) at the catalytic (**a**,**e**,**i**) and allosteric (**c**,**g**,**k**) binding sites of hMAO-A, respectively. 2D-binding diagrams showing the ligand–enzyme interactions for tanshinone I, tanshinone IIA, and cryptotanshinone at the catalytic (**b**,**f**,**j**) and allosteric (**d**,**h**,**l**) sites of hMAO-A, respectively.

**Figure 5:**
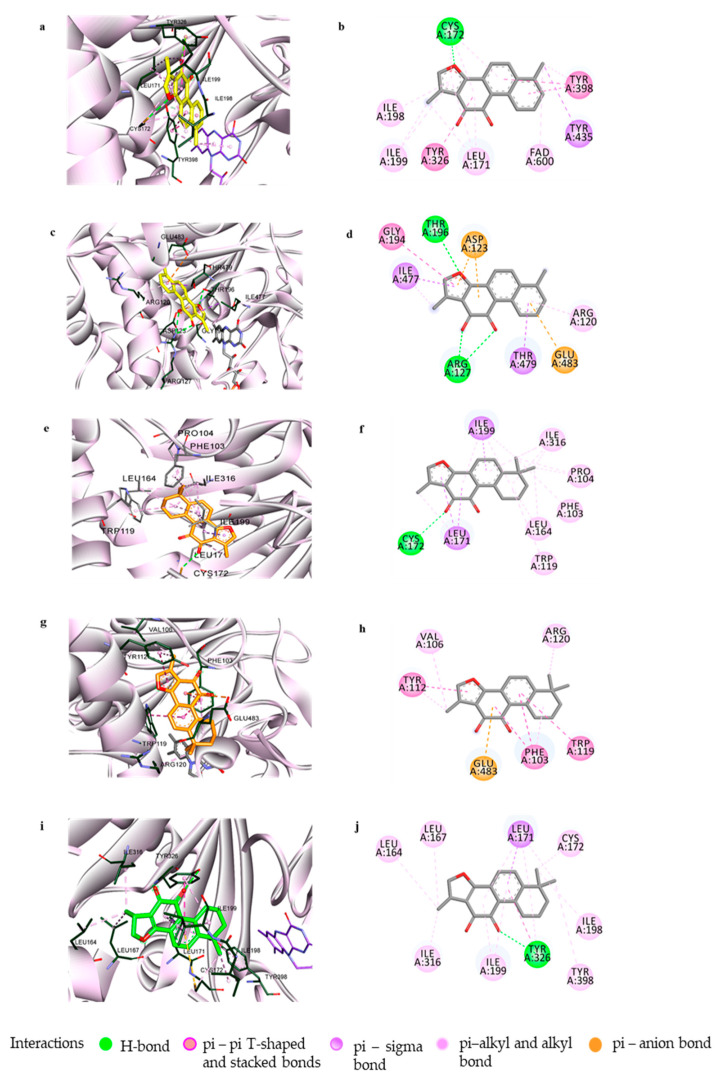
Prediction of the binding modes of the tanshinones with hMAO-B by molecular docking. Close-up view of the compounds tanshinone I (yellow), tanshinone IIA (brown), and cryptotanshinone (green) at the catalytic (**a**,**e**,**i**) and allosteric (**c**,**g**) binding sites of hMAO-B, respectively. 2D-binding diagrams showing the ligand–enzyme interactions for tanshinone I, tanshinone IIA, and cryptotanshinone at the catalytic (**b**,**f**,**j**) and allosteric (**d**,**h**) sites of hMAO-B, respectively.

To comprehend the specific binding sites and interactions at the orthosteric and/or allosteric sites of the enzymes responsible for the potent inhibitory actions of compounds **1**, **2**, and **3**, docking simulations were performed using Autodock4.2 software. hMAO-A (2BXR) was docked with the reference standard harmine, and hMAO-B (2BYB) was docked with the reference hMAO-B inhibitor deprenyl, to validate the docking results. Figure 4 and Figure 5 show the overall computational simulation results obtained at the best-docked pose for the compounds and MAO-A/B complexes. Table S2 presents the binding energies and the interacting residues in the enzyme-compound complexes.

As shown in Figure 4, tanshinones **1**, **2**, and **3** occupied the active site of hMAO-A and interacted with the catalytic binding site with low binding energies of –10.06, –9.91, and –10.07 kcal/mol, respectively. The 1-methylbenzofuran ring of **1** was aligned toward the catalytic aromatic cage comprising Tyr407, Tyr444, and the flavin adenine dinucleotide (FAD) cofactor of hMAO-A and interacted with these residues and FAD through strong hydrophobic bonds, such as π-π stacking, π-sigma, and π-alkyl bonds. The *O*–atom at C11 further associated with the FAD through a polar H-bond. The tyrosine residues surrounding FAD, Tyr444, and Tyr407 were considered recognition residues crucial for stable substrate/inhibitor binding through π-π interactions between the aromatic rings, while Ile335 and Phe208 of the active site were responsible for substrate selectivity [40,41,42]. The 1-methylnaphthalene moiety of **1** further linked with the substrate-specific residues Ile335 and Phe208 with aromatic π-sigma and π-π stacking interactions. The other active site amino acid residues in the ligand–enzyme complex formation were Ile180, Ile35, and Leu337. Compounds **2** and **3**, which differed in the 1,2-ene group in their benzofuran ring structures, faced towards the important catalytic site constituting FAD, Tyr407, and Tyr444 through their 6,6-dimethylnaphthalene moiety. Interactions with these catalytic residues, together with Phe352 and Tyr69, occurred via two methyl substituents, while the 1-methylbenzofuran-10,11-dione moiety participated in interactions with many active site residues of hMAO-A, such as Ile335, Ile325, Leu337, Ile 180, Cys323, Phe208, Val210, and others, as shown in Figure 4f,j.

Additionally, **1**, **2**, and **3** could be positioned in the allosteric binding sites of the hMAO-A enzyme, with similar orientations and binding energies of –7.87, –7.3, and –8.67 kcal/mol, respectively. Interactions with amino acid residues such as His488, Phe112, Trp128, Tyr121, and Tyr124 through hydrophobic bonds were common in these three compounds (Figure 4d,h,l). Compounds **1** and **2**, which have the same 1-methylnaphtho[1,2-g]furan-10,11-dione moiety, were anchored with identical residues of allosteric sites—His488 and Thr208 through the 1-CH_3_ and 11-*O* groups. All three compounds also showed an electrostatic π-anion association with Glu492.

Even though the tanshinones **1**, **2**, and **3** showed their most efficacious activity in hMAO-A inhibition, these compounds can also suppress hMAO-B significantly, as indicated by their IC_50_ values. To discover the structural activity relationship for the potent activity and selectivity of tanshinones in repressing hMAOs, we conducted a computational study of the compounds with hMAO-B. The molecular docking of **1** with hMAO-B involved interactions with catalytic recognition site residues Tyr398 and Tyr435 and the cofactor FAD600, which were considered vital for the stable inhibition of the enzyme, through its 6-methylnaphthalene group (Figure 5a,b). hMAO-B consisted of two cavities, one substrate cavity and a second hydrophobic cavity adjacent to the substrate cavity that separated the active site and the entrance cavity of hMAO-B. The compound also connected hydrophobically with the residues Cys172, Leu171, Tyr326, Ile199, and Ile198, present in the second cavity of hMAO-B. In addition, the docking study revealed that compound **1** was involved in allosteric site binding through different types of interactions—polar H-bonding with Thr196 (through 3-*O*) and Arg127 (through 10,11-*O*); electrostatic bonding with Asp123 (by the *o*-quinone moiety) and Glu483 (via 6-methylbenzene ring); and hydrophobic π-π stacking, π-π T-shaped interaction, and alkyl and π-alkyl bonding to Gly194, Ile477, Arg120, and Thr479.

Compound **2** also exhibited interactions with both the catalytic and allosteric site residues of hMAO-B with binding energies of −10.29 and −7.98 kcal/mol, respectively. As with **1**, the compound could anchor with Cys172 via a H-bond to the 11-*O* group and with Leu199 and Leu171 via hydrophobic interactions. From the binding pose analysis of **2** at the catalytic site, it was observed that the 6,6-dimethylcyclohexane ring projected outside the entrance cavity and interacted with the allosteric site residues Pro104, Phe103, Trp119, and Leu164 (Figure 5e,f). The in silico simulation also revealed that **2** can bind to the amino acid residues Val106, Tyr112, Arg120, and Glu483 of the allosteric site (Figure 5g,h). The docking revealed that the 1-methylfuran moiety of **2** could orient in two ways, either facing toward the catalytic site or toward the allosteric region, making it comparatively capable of binding either way. Compound **3** interacted with known catalytic site residues of the enzyme, such as Leu171, Ile198, Ile199, Tyr316, Cys176, Ile316, and Tyr398, along with Leu164 and Leu167 (Figure 5i,j).

### 3.3. In Silico Target Prediction of Tanshinones ***1***, ***2***, and ***3***

PCM is a computational technique that combines information from the ligand and related targets within a single machine learning model to explore the bioactivity of compounds on multiple related protein targets simultaneously. PCM is a quantitative biomodeling technique that can predict the affinity and selectivity of compounds across a panel of targets [43,44,45]. Our study used PCM to identify the possible targets for compounds **1**, **2**, and **3**. Table 2 lists the top 10 predicted targets for the compounds, ranked in the order of their normalization rate (NR). We selected muscarinic acetylcholine receptors for further experiments from the list of protein targets, since these GPCR receptors are associated with NDDs. Only compound **1** showed a likelihood of modulating muscarinic receptors.

### 3.4. Muscarinic Acetylcholine M_4_ Receptor Antagonist Action of Tanshinone I and the Molecular Docking Study

A functional GPCR assay that was conducted by measuring the cAMP, and Ca^++^ mobilization showed the selective antagonist nature of **1** on mAChR M_4_ (Table 3). The antagonist activity of **1**, represented by a percentage of the inhibition of the control agonist response, was found to be 56.1% ± 2.40% at 100 µM.

**Figure 6:**
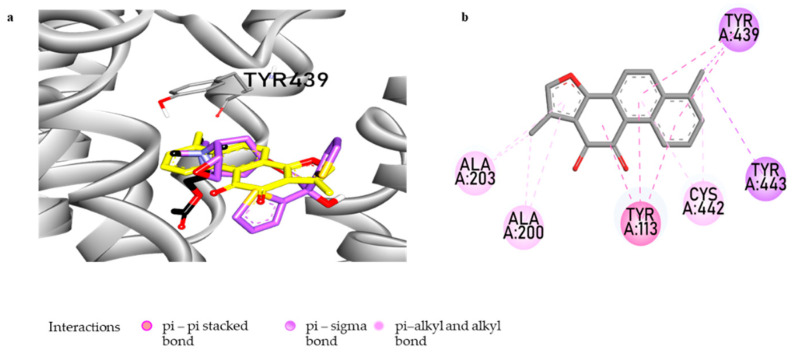
Prediction of the binding modes of tanshinone I with hM_4_R by molecular docking. Close-up view of tanshinone I at the orthosteric binding site of hM_4_R (**a**). Tanshinone-I, acetylcholine, and tiotropium are shown as yellow, black, and purple sticks, respectively. 2D-binding diagram showing ligand–receptor interactions at the catalytic hM_4_R by tanshinone I (**b**).

A computational study was used to investigate how the compound interacts with the M_4_ receptor (M_4_R) and at what target site the compound binds. The results of the molecular docking study, including the interacting residues and the binding energies, are presented in Figure 6 and Table S3. The docking pose analysis revealed that compound **1** occupies the orthosteric binding site of M_4_R and interacts with the amino acid residues of transmembranes (TM) 3, 5, and 7. Compound **1** binds with the catalytic site residues Tyr439 and Tyr433 via π-σ bonds, similar to the cognate agonist ACh. Interactions with Ala200 and Ala203 occur via π-alkyl bonds, similar to the cognate inverse agonist tiotropium. Additionally, compound **1** interacts with the active site residues Tyr113 via π-π stacking and Cys442 via π-alkyl and alkyl bonds. Unlike the reference ligands ACh and tiotropium, H-bonds and electrostatic interactions were not observed in Compound **1** and the M_4_R complex. Aromatic residues Tyr113 (TM3), Tyr416 (TM6), Tyr439 (TM7), and Tyr443 (TM7) have been linked to modulating the dissociation of antagonists from the orthosteric binding site of M_4_R. The mutations of these residues were found to reduce the binding affinity significantly. Moreover, the interactions of tiotropium with Asp112, Ser85, Tryp108, Tyr439, and Tyr443 were found specifically in M_4_R [46], suggesting that interactions with these residues are important for cooperativity in the ligand–M_4_R complex.

### 3.5. Prediction of the Pharmacokinetics and Toxicity Profile of Tanshinone I

The prediction of the pharmacokinetic profile for **1** by SwissADME showed a drug-likeness but no lead-likeness due to the violation of a condition where XLOGP3 should be less than 3.5 [47]. It was predicted to have a lipophilicity of 2.44 (log Po/w) and solubility of −6.91 (poorly soluble). The pkCSM application indicated high HIA (>90%), along with the probability to readily cross the BBB and reach the CNS for **1**. The pkCSM showed no toxicity on hepatocytes but is likely to be positive in the Ames test (Table 4).

## 4. Discussion

Herbal medicines have been used in TCM for numerous ailments in China and other Asian countries for thousands of years. The roots of *S. miltiorrhiza* are a widely popular component of TCM used either alone or combined with other herbs; for instance, the Danshen dripping pill (known as Fufang Dansen Diwan in China) is composed of *S*. *miltiorrhiza*, *Panax notoginseng,* and *Dryobalanops aromatica* [15]. The phytochemicals of Dansen have been clinically proven to possess multiple health-promoting effects, especially as cardiovascular and anticancer agents [48,49]. The neurological effects of *S*. *miltiorrhiza*, such as anti-Alzheimer’s, through the inhibition of cholinesterase [20], β-secretase [16], and amyloid β (Aβ) aggregation [50]; the attenuation of brain edema and protection of the BBB [51]; and the protection of dopaminergic neurons against neurotoxins [22,25], have highlighted the potential of its constituents in neurological disorders.

Our study investigated the hMAO isoenzyme inhibition potential of three abundant tanshinones of *S*. *miltiorrhiza*, supplemented with enzyme kinetics and molecular docking studies. Our research found that the test compounds were more active against hMAO-A than hMAO-B, consistent with a previous report [27]. Among the tested compounds, **1** showed the most potent and selective hMAO-A inhibition, with an IC_50_ value of 2.62 ± 0.52 µM and *K*_i_ value of 1.69 ± 0.19 µM. It showed a mixed-mode of enzyme inhibition, corroborating with catalytic and allosteric site binding in the docking studies. These results signify that **1** can bind to both free and substrate-bound hMAO-A enzymes. Compounds **2** and **3** also exhibited a strong mixed-mode of hMAO-A inhibition, with IC_50_ values of 6.08 ± 0.06 and 8.70 ± 0.06 µM and *K*_i_ values of 0.72 ± 0.13 and 4.99 ± 0.34 µM, respectively. The potency of the hMAO-A inhibition obtained in our work differs from that of a previously published article [27], because a lower concentration of these compounds was required to inhibit 50% of the enzyme activity. This variation might be because of the different experimental conditions and enzymes used.

Although tanshinones **1**, **2**, and **3** show selectivity toward hMAO-A, the ability of the compounds to suppress hMAO-B cannot be neglected, because they displayed enzyme inhibition at concentrations below 25 µM. MAO-B accounts for 80% of the total MAO activity and major dopamine oxidation in the striatum of the human brain compared to MAO-A. Moreover, MAO-B increases with aging and neurodegenerative diseases such as PD and AD, even though the age-related decline of many neurons and related neurotransmitters and enzymes occurs [52,53,54,55]. Juxtaposing the efficacy of the tested tanshinones for various functionalities related to NDDs, tanshinones **1**, **2**, and **3** were selective and notable inhibitors of hMAO-A, with moderate action against hMAO-B. The inhibition potential of these compounds was more significant for BChE than for AChE, suggesting their selectivity toward BChE [21]. Among the tested compounds, **1** was portrayed as the most efficacious for inhibiting BACE1 [16] and Aβ aggregation [50].

The in silico study revealed that the 1-methylbenzofuran ring with a single methyl substitution at C6 found in **1** was necessary for the strong π-π interactions with the critical catalytic site residues of hMAO-A. The 6,6-dimethyl substitution oriented the molecules in such a way that the methyl groups interacted with the Tyr444, Tyr407, and FAD600, preventing the aromatic sandwich interaction between the compounds and the enzyme, which is important for potent enzyme inhibition. Likewise, docking with hMAO-B showed that the structural features of **1** allowed it to interact with catalytic substrate-binding sites comprising FAD600, Tyr398, and Tyr 435, along with the second cavity residues, through polar and nonpolar bonding. In accord with the kinetic study, the inhibition mechanism established by the computational study showed catalytic and allosteric inhibition for **1** at the hMAO-B binding sites. The noncompetitive hMAO-B inhibitor **2**, on the other hand, was found to bind to both the catalytic and allosteric site residues to a similar degree. Similarly, **3** was engaged in the active site of hMAO-B interacting with most of the known second cavity loop residues and one substrate residue, Tyr398, of hMAO-B, which complied with its competitive binding mode.

Besides selective hMAO-A inhibition, tanshinones can target other proteins and enzymes. Among the high NR score targets, mAChRs have been linked to nondopaminergic motor and nonmotor symptoms of PD, and the only compound presumed to modulate these receptors through the PCM was **1**. The cell and nuclear receptor-based functional assay showed **1** to be a selective M_4_ antagonist. The in silico study revealed that the compound occupies the orthosteric binding site of the M_4_ receptor through hydrophobic interactions with active site residues, which were previously described by Thal et al. (2016) [46].

Although several studies have demonstrated the pharmacological benefits of tanshinones, the pharmacokinetics study showed a low bioavailability due to their low solubility and permeability. The in silico prediction of the pharmacokinetic characteristics of **1** revealed a poor solubility and low permeability (Caco-2 permeability was only 1.40 cm/s). As predicted for the CNS and BBB permeability, a recent in vivo pharmacokinetic and tissue distribution study using rats showed the distribution of **1** into the brain, with the highest amount reaching the liver, kidney, and lungs after oral administration. It was also observed that combinations of tanshinones had greater bioavailability (represented by the area under the curve and maximum concentration values) than single tanshinones administered orally, except for **1** and Dtan I, which showed similar pharmacokinetic values compared to that of the tanshinone mixture. Among tanshinones **1**, **2**, **3**, and Dtan I, **1** had the most extended half-life [56]. The ability of **1** to protect from hepatotoxicity was reported by Park et al. (2009), which conformed to the predicted non-hepatotoxic property [22,57]. Though the prediction showed **1** to be Ames-positive, indicating its mutagenic ability, its anticancer effects, together with its neuroprotective and anti-inflammatory effects, have been verified experimentally [22,50,58,59].

Neuropathological investigations in the autopsied brain of patients with PD have indicated that the disease manifests as dopaminergic neuronal degeneration in the ventrolateral substantia nigra in the early stage, which progresses to the midbrain and other regions of the brain in the later stages, along with increased levels of MAO-A and MAO-B [2,54]. Since DA is the common substrate for both MAO isoenzymes, the inhibitor of these enzymes prevents the oxidative degradation of DA and increases the synaptic DA level. The globally elevated MAO-A is correlated with mood disturbances such as sadness or depression in different psychiatric diseases and prodromal states and, therefore, represents a pharmacological target for correcting mood and depressive illnesses [54]. In addition to the dopaminergic system, the cholinergic system has been implicated in the etiology of the disease and is one of the pharmacological targets for managing the motor symptoms associated with PD.

M_4_ is the major mAChR subtype in the striatum, where it is enriched in the D_1_ DA receptor-expressing spiny projection neurons (SPNs) comprising the basal ganglia direct pathway (D_1_-SPNs) but not in D_2_ expressing indirect pathway SPNs. This is a critical pathway for motor activation. Studies using M_4_ deletion from D_1_-SPNs have found genetically modified mice to be hyperlocomotive, with elevated baseline DA, and more sensitive to dopaminergic stimulants than their littermate controls [60]. Tzavara et al. (2004) demonstrated that M_4_ muscarinic receptors regulate the dynamic balance between cholinergic and dopaminergic systems. In the M_4_ knockout mouse model, it was observed that the loss of M_4_ receptors enhanced the DA basal levels and induced dopaminergic hyperexcitability in response to psychostimulants, suggesting that M_4_ receptors are crucial muscarinic autoreceptors that regulate DA neurotransmission and dopaminergic activity [61]. Thus, selective M_4_R antagonists could improve a parkinsonian motor disability by relieving the M_4_-mediated inhibition of DA elevation and hyperexcitation of the dopaminergic system in the striatum. 

In former investigations that focused on the implications of diterpenoids from *S. miltiorrhiza* in AD, tanshinone congeners appreciably attenuated the scopolamine-induced cognitive impairments and reversed learning and memory dysfunctions induced by scopolamine and diazepam. These observations indicated that the memory-enhancing effect of tanshinones might be associated with the cholinergic signaling activation or the inhibition of cholinesterases that led to increased acetylcholine levels [62]. Although the cholinesterase inhibition properties of tanshinones **1**, **2**, and **3** are known, and the nAChR antagonist effects of the lipophilic extract of *S. miltiorrhiza* have been reported, whether the cognitive improvement by these tanshinone congeners is directly linked to cholinergic signaling remains unknown [20,21,62,63]. In PCM target prediction, we found that the muscarinic M_2_ and M_4_ receptors are the most relevant targets for **1**, and the in vitro experiment determined the M_4_ selective antagonist property of **1**, which might rule out the role of tanshinones in the stimulation of muscarinic receptors for learning and memory. Kim et al. (2009) found that the activation of the ERK/CREB signaling pathway by **1** in the hippocampus reversed GABA_A_ receptor agonist- and NMDA receptor antagonist-induced cognitive dysfunctions [64].

Due to the extreme similarity of the conserved residues that comprise the orthosteric sites of muscarinic receptors (M_1_–M_4_), designing a drug to act selectively at one of the mAChRs is challenging. Since M_4_ represents a crucial target for PD and selective M_4_ antagonists could produce therapeutic benefits, avoiding unwanted cholinergic side effects, the discovery of safe and novel M_4_-selective antagonists is vital. To date, a few M_4_-selective antagonists are known, and most clinically available antimuscarinic agents are either nonselective or only partially selective [46]. Our present work identifies that compound **1** from *S*. *miltiorrhiza* is a fully selective antagonist of M_4_. The investigation into the monoamine oxidase inhibitory activity of the compounds led to the acknowledgment that **1** is a potent and selective hMAO-A inhibitor with a mixed-mode of enzyme inhibition. The other tanshinones, **2** and **3**, also showed significant and selective hMAO-A inhibition with moderate activity against hMAO-B. Among the three tanshinones, **1** exhibited the most desirable pharmacological activities, along with the reported abilities to inhibit cholinesterases, BACE1, and Aβ aggregation, suggesting that **1** may have a promising role in neurodegenerative diseases. Overall, our present in vitro study revealed compound **1** as a potent hMAO-A/B inhibitor and a selective M_4_R antagonist. These bioactivities suggest the potential therapeutic benefits of **1** in alleviating DA deficiency, motor symptoms, and depression in NDDs. However, further in vivo investigations are important to establish **1** as a potential M_4_R antagonist and a MAO inhibitor to treat motor dysfunctions and depression.

## Figures and Tables

**Table 1:** Inhibitory activities of tanshinone I, tanshinone IIA, and cryptotanshinone against recombinant human monoamine oxidase A and B and their enzyme kinetic parameters.

Compound	Human Monoamine Oxidase A (hMAO-A)				SI *^b^*	Human Monoamine Oxidase B (hMAO-B)			
	IC_50_ *^a^*	*K*_ic_ *^c^*	*K*_iu_ *^c^*	Inhibition Type *^d^*		IC_50_ *^a^*	*K*_ic_ *^c^*	*K*_iu_ *^c^*	Inhibition Type *^d^*
Tanshinone I	2.62 ± 0.52	1.69 ± 0.19	4.65 ± 0.16	Mixed	0.11	24.9 ± 3.82	25.6 ± 1.10	17.4 ± 0.78	Mixed
Tanshinone IIA	6.08 ± 0.06	0.72 ± 0.13	2.74 ± 0.37	Mixed	0.35	17.5 ± 0.89	12.9 ± 1.14	13.7 ± 0.58	Non-competitive
Cryptotanshinone	8.70 ± 0.06	4.99 ± 0.34	38.1 ± 1.79	Mixed	0.38	23.1 ± 2.10	9.33 ± 0.10	-	Competitive
l-Deprenyl·HCl *^e^*	14.9 ± 0.38	-	-	-	78.8	0.19 ± 0.02	-	-	-‘
Clorgyline.HCl *^f^*	0.008 ± 0.00	-	-	-	-	-	-	-	-

*^a^* The concentration required to produce a 50% inhibition of the hMAO-A/B activities (IC_50_ values in µM) was calculated using the log-dose inhibition curve and expressed as the mean ± SD of triplicate experiments. *^b^* Selectivity index (ratio of the IC_50_ value for hMAO-A inhibition to that for hMAO-B inhibition). *^c^* The hMAO inhibition constants (*K*_ic_ and *K*_iu_ values in μM) were determined from the secondary plots. *^d^* The hMAO inhibition type was determined using Lineweaver-Burk and Dixon plots. *^e,f^* Positive controls.

**Table 2:** List of the top ten protein targets for tanshinone I, tanshinone IIA, and cryptotanshinone from computational proteochemometric modeling.

Tanshinone I	NR *^a^*	Tanshinone IIA	NR *^a^*	Cryptotanshinone	NR *^a^*
Gastrin/cholecystokinin type B receptor	0.96	Mitogen-activated protein kinase 14	0.85	Mitogen-activated protein kinase 14	0.95
Somatostatin receptor type 2	0.95	Vascular endothelial growth factor receptor 1	0.73	Mitogen-activated protein kinase 8	0.85
Endothelin-1 receptor	0.94	Mitogen-activated protein kinase 8	0.71	Hepatocyte growth factor receptor	0.74
Muscarinic acetylcholine receptor M_4_	0.92	Vasopressin V1b receptor	0.70	Vasopressin V1b receptor	0.73
Muscarinic acetylcholine receptor M2	0.92	Hepatocyte growth factor receptor	0.67	Prostaglandin G/H synthase 2	0.68
B1 bradykinin receptor	0.92	Sodium-dependent serotonin transporter	0.65	Vascular endothelial growth factor receptor 1	0.68
Histamine H1 receptor	0.92	RAC-beta serine/threonine-protein kinase	0.61	Type-1 angiotensin II receptor	0.67
5-hydroxytryptamine receptor 2A	0.92	Phosphatidylinositol 4,5-bisphosphate 3-kinase catalytic subunit alpha isoform	0.61	Vitamin D3 receptor	0.67
Type-1 angiotensin II receptor	0.92	Proto-oncogene tyrosine-protein kinase Src	0.61	Receptor tyrosine-protein kinase erbB-2	0.67
Beta-1 adrenergic receptor	0.91	RAC-alpha serine/threonine-protein kinase	0.61	RAC-beta serine/threonine-protein kinase	0.67

*^a^* NR: normalization rate.

**Table 3:** Percentage stimulation and percentage inhibition of recombinant human muscarinic acetylcholine receptors by tanshinone I.

Receptors	% Stimulation *^a^*	% Inhibition *^b^*	Reference Agonist *^c^* (Reference Antagonist) *^d^*	Reference EC_50_ *^e^* (IC_50_) *^f^*
M_1_	2.5 ± 0.21	13.8 ± 1.98	Acetylcholine (Pirenzepine)	0.6 (49)
M_2_	12.6 ± 3.61	35.8 ± 4.74	Acetylcholine (Methoctramine)	70 (140)
M_3_	−2.3 ± 0.21	3.3 ± 11.24	Acetylcholine (4-DAMP)	27 (4.1)
M_4_	−23.5 ± 1.48	56.1 ± 2.40	Acetylcholine (PD 102807)	26 (36)
M_5_	−1.6 ± 0.85	−0.5 ± 0.42	Acetylcholine (Atropine sulfate)	2 (2.1)

*^a^* % stimulation represents the percentage of the control agonist response by tanshinone I at 100 µM. *^b^* % inhibition represents the percentage inhibition of the control agonist response by tanshinone I at 100 µM. *^c^* Reference agonists and *^d^* reference antagonists used in the assay. *^e^* EC_50_ value of reference agonist (nM). *^f^* IC_50_ value of reference antagonist (nM). 4-DAMP: 1,1-Dimethyl-4-diphenylacetoxypiperidinium iodide.

**Table 4:** Prediction of the pharmacokinetic parameters and toxicity of tanshinone I.

Parameters	Tanshinone I
Drug-likeness	Yes
Lead-likeness	No; 1 violation: XLOGP3 > 3.5
Log Po/w *^a^*	2.44
Solubility *^b^*	−6.91
HIA *^c^*	98.91%
Caco-2 permeability *^d^*	1.401
BBB permeability *^e^*	Yes (0.447)
CNS permeability *^f^*	−1.446
AMES toxicity	Yes
Hepatotoxicity	No

*^a^* Log of the coefficient for solvent partitioning between 1-octanol and water. *^b^* LogS scale: Insoluble < −10 < Poorly < −6 < Moderately < −4 < Soluble < −2 < Very < 0 < Highly.*^c^* Human intestinal absorption: If <30%, poorly absorbed. *^d^* Caco-2 permeability is high if it has Papp > 8 × 10^−6^ cm/s. *^e^* Log BB > 0.3 was considered to readily cross the blood–brain barrier, while log BB < −1 was considered to be poorly distributed to the brain. *^f^* Log PS > −2 was considered to penetrate the central nervous system (CNS), while log PS < −3 was considered unable to penetrate the CNS.

## Data Availability

The data relevant to this study are provided in the manuscript and supplementary information. Other additional information can be obtained from the corresponding authors upon reasonable request.

## Supplementary Materials

The following are available online at https://www.mdpi.com/article/10.3390/biom11071001/s1. The Supplementary Information for this paper is available as Table S1. In vitro pharmacology: Cellular and nuclear receptor functional assays, Table S2. Binding site residues and docking scores of tanshinone I, tanshinone IIA, cryptotanshinone, and the reference ligands in the human monoamine oxidases (hMAO-A and hMAO-B), Table S3: Binding site residues and docking scores of tanshinone I and the reference ligands in the human M_4_ muscarinic acetylcholine receptor (hM_4_R).
