# Supplementary material for: Monoamine Oxidase Inhibition by Major Tanshinones from Salvia miltiorrhiza and Selective Muscarinic Acetylcholine M4 Receptor Antagonism by Tanshinone I
Source: Biomolecules. 2021 Jul 8;11(7):1001. doi: 10.3390/biom11071001 (PMC8301926; doi:10.3390/biom11071001)
Supplement: Supplementary file 1 [file biomolecules-11-01001-s001.zip › biomolecules-1247719-supplementary.pdf]

SUPPLEMENTARY INFORMATION

Article

Monoamine Oxidase Inhibition by Major Tanshinones from *Salvia miltiorrhiza* and Selective Muscarinic Acetylcholine M_4 Receptor Antagonism by Tanshinone I

Ritu Prajapati [1], Se Eun Park [1,2], Su Hui Seong [1,3], Pradeep Paudel [1,4], Fazlin Mohd Fauzi [5], Hyun Ah Jung [6,*] and Jae Sue Choi [1,*]

[1] Department of Food and Life Science, Pukyong National University, Busan 48513, Korea; ritpraz@gmail.com (R.P.), gogo1685@mail.ulsan.ac.kr (S.E.P.); shseong@hnibr.re.kr (S.H.S.); ppradeep@olemiss.edu (P.P.)

[2] Department of Biomedical Science, Asan Medical Institute of Convergence Science and Technology, University of Ulsan, Seoul 05505, Korea

[3] Natural Product Research Division, Honam National Institute of Biological Resource, Mokpo 58762, Korea

[4] National Center for Natural Products Research, Research Institute of Pharmaceutical Science, The University of Mississippi, Oxford, MS 38677, USA

[5] Department of Pharmacology and Chemistry, Faculty of Pharmacy, Universiti Teknologi MARA, Selangor 42300, Malaysia; fazlin5465@uitm.edu.my

[6] Department of Food Science and Human Nutrition, Jeonbok National University, Jeonju 54896, Korea

* Correspondence: choijs@pknu.ac.kr (J.S.C.); jungha@jbnu.ac.kr (H.A.J.); Tel.: +82-51-629-7547 (J.S.C.); +82-63-270-4882 (H.A.J.)

Contents

Table S1 ... 3

Table S2 ... 8

Table S3 ... **Error! Bookmark not defined.**

References ... 9

Table S1. In vitro pharmacology: Cellular and nuclear receptor functional assays.

Assay	Source	Stimulus	Incubation	Measured Component	Detection Method	References
M_1 (h) (agonist effect)	Human recombinant CHO cells	None (100 nM acetylcholine for control)	RT	Intracellular [Ca^{2+}]	Fluorimetry	[33]
M_1 (h) (antagonist effect)	Human recombinant CHO cells	Acetylcholine (10 nM)	RT	Intracellular [Ca^{2+}]	Fluorimetry	[33]
M_2 (h) (agonist effect)	Human recombinant CHO cells	None (3 µM acetylcholine for control)	10 min 37 °C	cAMP	HTRF	[34]
M_2 (h) (antagonist effect)	Human recombinant CHO cells	Acetylcholine (300 nM)	10 min 37 °C	cAMP	HTRF	[34]
M_3 (h) (agonist effect)	Human recombinant CHO cells	None (1 µM acetylcholine for control)	RT	Intracellular [Ca^{2+}]	Fluorimetry	[33]
M_3 (h) (antagonist effect)	Human recombinant CHO cells	Acetylcholine (100 nM)	RT	Intracellular [Ca^{2+}]	Fluorimetry	[33]
M_4 (h) (agonist effect)	Human recombinant CHO cells	None (1µM acetylcholine for control)	10 min 37 °C	cAMP	HTRF	[35]
M_4 (h) (antagonist effect)	Human recombinant CHO cells	Acetylcholine (100 nM)	10 min 37 °C	cAMP	HTRF	[35]
M_5 (h) (agonist effect)	Human recombinant RBL cells	None (0.624 µM acetylcholine for control)	RT	Intracellular [Ca^{2+}]	Fluorimetry	[36]
M_5 (h) (antagonist effect)	Human recombinant RBL cells	Acetylcholine (10 nM)	RT	Intracellular [Ca^{2+}]	Fluorimetry	[36]

HTRF: homogeneous time-resolved fluorescence; RT: room temperature

Table S2. Binding site residues and docking scores of tanshinone I, tanshinone IIA, cryptotanshinone and the reference ligands in human monoamine oxidases (hMAO-A and hMAO-B).

Ligand	Binding Energy (kcal/mol)[a]	Interacting residue[b]		
		H-bond	Hydrophobic	Electrostatic
		hMAO-A		
Tanshinone I (catalytic inhibitor)	-10.06	FAD600	Pi-sigma: Ile335, Tyr407, FAD600, Phe208<br>Pi-Pi Stacked: Tyr407, FAD600, Ile335<br>Alky: Ile335<br>Pi-Alkyl: Tyr444, FAD600, Ile180, Ile35, Leu337	–
Tanshinone I (allosteric inhibitor)	-7.87	Thr205	Pi-sigma: His488<br>Pi-Pi T-shaped: Phe112, Trp128<br>Alkyl: Arg129<br>Pi-Alkyl: Tyr121, Tyr124, Arg129	Pi-Anion: Glu492
Tanshinone IIA (catalytic inhibitor)	-9.91	–	Pi-sigma: Ile335, Tyr407<br>Pi-Pi T-shaped: Phe208<br>Alkyl: Cys323, Ile335, Ile325<br>Pi-Alkyl: Tyr69, Phe208, Phe352, FAD600, Leu337, Ile180, Ile335	–

Tanshinone IIA (allosteric inhibitor)	-7.3	Thr205	Pi-sigma: His488<br>Pi-Pi T-shaped: Phe112, Tyr124, Trp128<br>Alkyl: Arg129<br>Pi-Alkyl: Tyr121, Tyr124, Arg129	Pi-Anion: Glu492
Cryptotanshinone (catalytic inhibitor)	-10.07	–	Pi-sigma: Tyr407<br>Pi-Pi T-shaped: Phe208<br>Alkyl: Val210, Cys323, Leu337<br>Pi-Alkyl: Tyr69, Phe352, Tyr407, FAD600, Ile180, Ile335	–
Cryptotanshinone (allosteric inhibitor)	-8.67	His488	Pi-Pi T-shaped: Phe112, Trp128<br>Alkyl: Val115<br>Pi-Alkyl: Tyr121, Tyr124, His488	Pi-Anion: Glu492
HRM[c]	-6.72	Asn181	Pi-Sigma: Tyr444<br>Pi-Pi Stacked: Tyr407<br>Alkyl: Ile180<br>Pi-Alkyl: Phe208, Tyr407, FAD600, Ile180, Ile335	–
		hMAO-B		

Tanshinone I<br>(catalytic inhibitor)	-9.87	Cys172	Pi-Sigma: Tyr435<br>Pi-Pi Stacked: Tyr398<br>Pi-Pi T-shaped: Tyr326<br>Alkyl: Leu171, Ile199<br>Pi-Alkyl: Tyr398, FAD600, Leu171, CYs172, Ile198, Ile199	–
Tanshinone I<br>(allosteric inhibitor)	-7.5	Arg172, Thr196	Pi-Sigma: Ile477, THr479<br>Amide-Pi Stacked: GLy194, THr195<br>Alkyl: Ile477<br>Pi-Alkyl: Arg120	Pi-Anion: Asp123, Glu483
Tanshinone IIA<br>(catalytic inhibitor)	-10.29	Cys172	Pi-Sigma: Leu171, Ile199<br>Alkyl: Ile199, Ile316, Pro104, Leu164, Leu171<br>Pi-Alkyl: Phe103, Trp119, Ile316, Ile199	–
Tanshinone IIA<br>(inhibitor)	-7.98		P-Pi Stacked: Phe103, Tyr112, Trp119<br>Alkyl: Trp119, Arg120, Val106<br>Pi-Alkyl: Phe103, Tyr112	Pi-Anion: Glu483

Cryptotanshinone (catalytic inhibitor)	-9.89	Tyr326	Pi-Sigma: Leu171<br>Pi-Pi T-shaped: Tyr326<br>Alkyl: Leu171, Cys172, Ile198, Leu164, Leu167, Ile316<br>Pi-Alkyl: Tyr326, Tyr398, Ile199, Leu171	–
Deprenyl[d]	-6.32	Leu171	Pi-Sigma: Leu171<br>Pi-Pi T-shaped: Tyr326<br>Pi-Alkyl: Phe343, Tyr398, FAD600, Ile199	–

[a]Estimated free binding energy of the ligand–receptor complex. [b]The number of hydrogen bonds and all amino acid residues from the enzyme–inhibitor complex were determined with the AutoDock 4.2 program. [c]7-Methoxy-1-methyl-9H-pyrido [3,4-b]indole. [d]Reference ligand.

Table S3. Binding site residues and docking scores of tanshinone I and the reference ligands in human M_4 muscarinic acetylcholine receptor (hM_4R).

Ligand	Binding Energy (kcal/mol)[a]	Interacting residue[b]		
		H-bond	Hydrophobic	Electrostatic and others
Tanshinone I	-8.77	–	Pi-Sigma: Tyr439, Tyr443<br>Pi-Pi stacked: Tyr113, Tyr439<br>Pi-Alkyl: Ala200, Ala203, Cys442<br>Alkyl: Ala200, Ala203, Cys442	–
Acetylcholine[c] (Agonist)	-3.92	Ser116	Pi-Sigma: Tyr439, Tyr433	Attractive charge: Asp112<br>Pi-Cation: Tyr439
Tiotropium[c] (Inverse agonist)	-10.12	Asn417	Pi-Sigma: Tyr439<br>Pi-Alkyl: Ala200, Val420, Ala203	Attractive charge: Asp112<br>Pi-Cation: Tyr439<br>Pi-Sulfur: Trp164

[a]Estimated free binding energy of the ligand–receptor complex. [b]The number of hydrogen bonds and all amino acid residues from the enzyme–inhibitor complex were determined with the AutoDock 4.2 program. [c]Reference ligands.

References [in the main text]

33. Sur, C.; Mallorga, P.J.; Wittmann, M.; Jacobson, M.A.; Pascarella, D.; Williams, J.B.; Brandish, P.E.; Pettibone, D.J.; Scolnick, E.M.; Conn, P.J. N-desmethylclozapine, an allosteric agonist at muscarinic 1 receptor, potentiates N-methyl-D-aspartate receptor activity. *Proc. Natl. Acad. Sci,* **2003**, *100*, 13674-13679.
34. Michal, P.; Lysíková, M.; Tuček, S. Dual effects of muscarinic M_2 acetylcholine receptors on the synthesis of cyclic AMP in CHO cells: dependence on time, receptor density and receptor agonists. *Br. J. Pharmacol.* **2001**, *132*, 1217-1228.
35. Olianas, M.C.; Onali, P. PD 102807, a novel muscarinic M_4 receptor antagonist, discriminates between striatal and cortical muscarinic receptors coupled to cyclic AMP. *Life Sci.* **1999**, *65*, 2233-2240.
36. Caulfield, M.P.; Birdsall, N.J. International Union of Pharmacology. XVII. Classification of muscarinic acetylcholine receptors. *Pharmacol. Rev.* **1998**, *50*, 279-290.
